# Practice of COVID-19 Preventive Measures and Its Associated Factors among Students in Ghana

**DOI:** 10.4269/ajtmh.20-1301

**Published:** 2020-12-07

**Authors:** Paschal Awingura Apanga, Isaac Bador Kamal Lettor, Ramatu Akunvane

**Affiliations:** 1 School of Community Health Sciences, University of Nevada, Reno, Nevada;; 2 Bawku Technical Institute, Bawku, Ghana;; 3 Nursing and Midwifery Training College, Zuarungu, Ghana

## Abstract

Preventive measures are key to reducing COVID-19 morbidity and mortality. Our primary aim was to assess factors associated with practice of COVID-19 preventive measures among senior high school students in the Bawku Municipality, Ghana. A cross-sectional study was conducted, and data on 624 students were analyzed using multivariable logistic regression. An estimated 31.5% (95% CI: 27.8, 35.1) of the students wore a face mask often or always. Students who reported that COVID-19 can be transmitted via droplets from the nose or mouth (adjusted odds ratios [aOR]: 3.0; 95% CI: 1.1, 7.9) and students who reported that asymptomatic persons can transmit the virus (aOR: 2.0; 95% CI: 1.2, 3.6) had higher odds of wearing a face mask. However, students who reported that COVID-19 was not deadly were associated with lower odds of wearing a face mask (aOR: 0.6; 95% CI: 0.4, 1.0). Handwashing/hand sanitizing was practiced by 49.5% (95% CI: 45.6, 53.5) of students. Students in the technical program compared to the science program (aOR: 0.4; 95% CI: 0.2, 0.8) and those who reported that COVID-19 was not deadly (aOR: 0.6, 95% CI: 0.4, 1.0) had lower odds of handwashing/hand sanitizing. An estimated 46.2% (95% CI: 42.3, 50.2) of students practiced social distancing. Students who reported that COVID-19 can be transmitted via droplets from the nose or mouth were positively associated with social distancing (aOR: 2.1; 95% CI: 1.0, 4.5). There is the need to intensify education about COVID-19 in senior high schools while enforcing the practice of COVID-19 preventive measures.

## INTRODUCTION

The COVID-19 pandemic, which was first reported in Wuhan, Hubei Province of China in December 2019 has become a global health crisis.^1,2^ The pandemic has led to increased COVID-19 morbidity and mortality.^3^ As of October 2, 2020, more than 34.6 million cases and one million deaths were reported globally.^4^ The pandemic has also had devastating effects on the world’s economy with collapse of many businesses and loss of many jobs.^5,6^ The educational sector has also been disrupted, with some schools adopting to new ways of teaching and learning.^7^

COVID-19 is a disease caused by a novel coronavirus named SARS-CoV-2.^8,9^ SARS-CoV-2 is a RNA virus, which is characterized by symptoms such as fever or chills, cough, difficulty breathing, fatigue, muscle or body aches, headache, new loss of taste or smell, sore throat, congestion or runny nose, nausea or vomiting, and diarrhea.^8,10^ Development of these symptoms may appear 2–14 days after exposure to the virus.^8,9^ Coronavirus can be transmitted via animal-to-human and human-to-human interactions.^9^ Whereas human-to-human transmission routes include droplet inhalation, coughing, and sneezing, contact transmission modes include feco-oral, nasal, and eye mucous membrane contacts.^11–13^ Practicing preventive measures such as handwashing with soap and water, wearing of face mask, social distancing, covering of the mouth and nose when coughing, and avoiding touching of the face can prevent transmission of COVID-19 infection.^14^

In Ghana, the first case of COVID-19 was reported on March 12, 2020.^15^ As of October 2, 2020, Ghana reported a total of 46,694 cases, with 301 deaths positioning it as the country with the seventh highest number of confirmed cases in Africa and 63rd globally.^4^ In response to the global pandemic, the government of Ghana adopted several control strategies to reduce the morbidity and mortality associated with COVID-19. Key among these strategies included testing, tracing, and treatment (“3 Ts”) approach; lockdowns in some parts of the country; and ensuring that all Ghanaians practice COVID-19 preventive measures.^16,17^ Despite these measures taken by the government of Ghana to control the COVID-19 pandemic, the WHO observed that Ghana is one of the countries with a substantial increase in the number of COVID-19 cases.^18^

As part of measures to control the pandemic in Ghanaian schools, the government of Ghana in June 2020 introduced a phased reopening of schools, commencing with final year students in tertiary institutions, and junior and senior high schools to enable them prepare for their final examination, while ensuring that they observe COVID-19 safety protocols.^19^ However, some students tested positive to COVID-19 after returning to school,^20^ which raises concern whether schools have implemented COVID-19 safety protocols. Despite the emphasis on students to practice COVID-19 preventive measures while in school, we do not know the factors associated with practicing COVID-19 preventive measures among students in Ghana. Our primary aim was to assess factors associated with the practice of COVID-19 preventive measures among senior high school students in Ghana.

## METHODS

### Study design, data collection, and study population.

This was a cross-sectional study conducted in the Bawku Municipality in the Upper East Region of Ghana. The Upper East Region is one of 16 regions of Ghana. Data were collected from final year students from four senior high schools in the Bawku Municipality. The municipality, schools, and students were conveniently sampled for the study. The inclusion criteria were students in any of the four selected schools who were aged 18 years and older. Data were collected by administering structured questionnaires to students in selected schools who met the inclusion criteria. Data were collected via self-administered questionnaires on August 3–7, 2020. The minimum sample size required for the study was estimated based on our assumption that 50% of students observed COVID-19 preventive measures. Using a 5% margin of error with a 95% CI, a minimum sample size of 384 was required. We however approached 631 students, but seven students were ineligible because they did not meet the inclusion criteria. The remaining 624 students participated and completed the questionnaires. The questionnaire consisted of questions on sociodemographics, knowledge about COVID-19 common symptoms, transmission, and preventive measures against COVID-19.

### Outcomes.

Our primary outcomes of interest included three key COVID-19 preventive measures. The following primary outcomes were measured: wearing of face mask, handwashing with soap and water or with alcohol-based hand sanitizer, and social distancing. Wearing of face mask was dichotomized as “1” for a student who always or often wears a face mask irrespective of the type of face mask and “0” for those who reported wearing the face mask sometimes or not at all. Handwashing with soap and water or hand sanitizing was also dichotomized as “1” for a student who always or often washes his/her hands with soap and water or sanitizes the hands with alcohol-based hand sanitizer^14^ and “0” for students who either do not wash/sanitize their hands or sometimes handwash/sanitize. Social distancing was defined as maintaining at least one meter distance between a student and others.^14^ It was dichotomized as “1” for students who practiced it always or often and “0” for students who did not practice it or practiced it sometimes.

### Predictor variables.

We explored variables of interest that included knowledge of COVID-19 symptoms, knowledge of COVID-19 mode of transmission, knowledge on COVID-19 preventive measures, and whether an educational/training program was a source of information about COVID-19.

With regard to knowledge of COVID-19 symptoms, we compared students who knew one or more symptoms of COVID-19 (fever, dry cough, body aches, tiredness, diarrhea, sore throat, nasal congestion, conjunctivitis, headache, loss of taste or smell, and rash on fingers or toes) with students who did not know any of the symptoms.

On mode of transmission of COVID-19, we compared students who reported that COVID-19 can be transmitted via droplets from the nose or mouth of an infected person with students who reported that this was not a transmission route. A comparison was also made on students who reported that COVID-19 can be transmitted by touching droplets on surface or objects from an infected person with students who reported that this was not a transmission route. We also compared students who reported that an infected asymptomatic person can transmit the virus with students who reported otherwise. Students who reported that COVID-19 can be transmitted among adolescents and young adults were also compared with students who reported that transmission was not possible.

On knowledge about COVID-19 preventive measures, we compared students who reported that avoiding crowdedness in public places can prevent infection with COVID-19 with students who reported that it was not a preventive measure. Students who reported that covering the nose and mouth while coughing can prevent COVID-19 were also compared with students who reported that it was not a preventive measure. We also compared students who reported that avoiding the touching of the eyes, nose, and mouth can prevent infection with COVID-19 with students who reported otherwise. Students who reported that COVID-19 was not deadly and there was no need for preventive measures were compared with students who reported that the virus was deadly.

Students who reported they had heard about COVID-19 via an educational or training program were compared with students who did not hear about it through an educational/training program.

### Covariates.

Covariates included age, gender (male and female), father’s level of education (no education, primary education, and secondary or higher education), mother’s level of education (no education, primary education, and secondary or higher education), has health insurance (yes or no), place of residence when school is not in session (rural and urban), and program of study (science, arts, business, technical, and home economics/catering/agricultural science). The program of study refers to the course the student is majoring in the school.

### Data analysis.

Data analysis was conducted using SAS version 9.3 (SAS Institute, Cary, NC). Descriptive statistics was used to report the characteristics of the study population. We used three multivariable logistic regression models to assess the relationship between our predictor variables of interest and each of our primary outcomes while controlling simultaneously for all our covariates. We obtained adjusted odds ratio (aOR) estimates for the predictor variables in each of the models. Our study protocol was approved by the Christian Health Association of Ghana Institutional Review Board. We also obtained informed consent from students before data collection as well as observing COVID-19 safety protocols.

## RESULTS

### Study sample.

The study population consisted of 624 students aged 18 years and older. The mean age of the study population was 19.9 years, with a SD of 1.48. A majority of the students were males (54.2%). Many of the students had a health insurance (78.5%), and most of them were in the technical program (38.0%), compared with other programs (Table 1).

**Table 1:** Characteristics of study population (*n* = 624)

Variable	*N* (%) or mean (SD)
Age (years)	19.9 (1.48)
Gender	
Male	338 (54.2)
Female	286 (45.8)
Mother’s education	
No formal education	381 (61.7)
Primary	143 (23.1)
Secondary or higher education	94 (15.2)
Father’s education	
No formal education	358 (58.0)
Primary	112 (18.2)
Secondary or higher education	147 (23.8)
Has health insurance	
No	134 (21.5)
Yes	490 (78.5)
Place of residence when school is not in session	
Rural	400 (64.5)
Urban	220 (35.5)
Program of study	
Science	32 (5.1)
Arts	146 (23.4)
Business	104 (16.7)
Technical	237 (38.0)
Home economics/catering/agricultural	105 (16.8)

**Figure 1.**
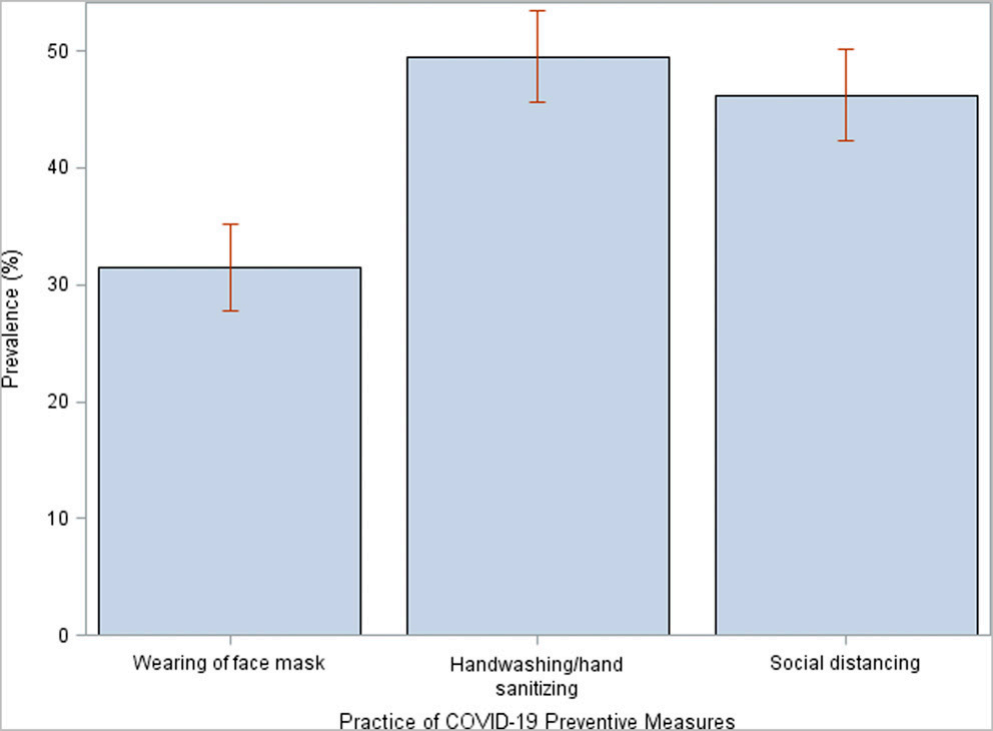
Bar plot showing the prevalence of practice of COVID-19 preventive measures among senior high schools in Ghana. This figure appears in color at www.ajtmh.org.

An estimated 31.5% (95% CI: 27.8, 35.1) of the students wore a face mask often or always; 49.5% (95% CI: 45.6, 53.5) of the students practiced handwashing or sanitized their hands with alcohol-based sanitizers often/always. The prevalence of social distancing among students was 46.2% (95% CI: 42.3, 50.2; Figure 1).

The multivariable logistic regression analyses showed that students who reported that COVID-19 can be transmitted via droplets from the nose or mouth of an infected person had three times the odds of wearing a face mask compared with students who reported that it was not transmitted via droplets from the nose or mouth of an infected person (aOR: 3.0; 95% CI: 1.1, 7.9). The odds of wearing a face mask among students who reported that asymptomatic persons with COVID-19 could transmit the virus was twice the odds of wearing a face mask among students who reported that asymptomatic persons cannot transmit the virus (aOR: 2.0; 95% CI: 1.2, 3.6). The odds of wearing a face mask among students who reported that COVID-19 was not deadly had a 40% lower odds of wearing a face mask than those who reported that COVID-19 was deadly (aOR: 0.6; 95% CI: 0.4, 1.0) (Table 2).

**Table 2:** Factors associated with wearing a face mask, handwashing/sanitizing, and social distancing

Variable	Wearing a face mask, aOR (95% CI)	Practicing handwashing/sanitizing, aOR (95% CI)	Practicing social distancing, aOR (95% CI)
Age (years)	1.0 (0.9, 1.2)	1.0 (0.9, 1.1)	1.1 (0.8, 1.1)
Gender			
Male	1	1	1
Female	1.0 (0.7, 1.5)	0.9 (0.6, 1.2)	1.2 (0.8, 1.7)
Mother's education			
No formal education	1	1	1
Primary	0.6 (0.4, 1.0)	0.7 (0.4, 1.0)	0.7 (0.4, 1.1)
Secondary or higher education	0.8 (0.4, 1.5)	1.1 (0.6, 2.0)	1.3 (0.7, 2.3)
Father's education			
No formal education	1	1	1
Primary	1.0 (0.6, 1.6)	1.1 (0.7, 1.8)	0.8 (0.5, 1.3)
Secondary or higher education	1.1 (0.7, 1.9)	0.7 (0.4, 1.2)	0.7 (0.4, 1.1)
Program of study			
Science	1	1	1
Arts	0.8 (0.3, 1.8)	0.9 (0.4, 2.0)	1.5 (0.7, 3.4)
Business	0.8 (0.3, 1.8)	0.7 (0.3, 1.6)	0.9 (0.4, 2.1)
Technical	0.5 (0.2, 1.2)	0.4 (0.2, 0.8)*	0.7 (0.3, 1.6)
Home economics/catering/agricultural	0.9 (0.4, 2.3)	0.9 (0.4, 2.0)	1.2 (0.5, 2.8)
Has health insurance			
No	1	1	1
Yes	1.1 (0.7, 1.7)	1.2 (0.8, 1.8)	1.0 (0.7, 1.6)
Place of residence when school is not in session			
Rural	1	1	1
Urban	1.1 (0.7, 1.6)	1.0 (0.7, 1.5)	0.8 (0.6, 1.2)
Knowledge of COVID-19 symptoms			
None	1	1	1
Knowing one or more symptoms	0.4 (0.1, 2.3)	0.6 (0.1, 2.7)	0.6 (0.1, 3.0)
COVID-19 can be transmitted via droplets from the nose or mouth			
No	1	1	1
Yes	3.0 (1.1, 7.9)*	1.9 (0.9, 3.9)	2.1 (1.0, 4.5) **
COVID-19 can be transmitted by touching droplets on surface or objects			
No	1	1	1
Yes	0.7 (0.3, 1.7)	0.5 (0.2, 1.1)	0.7 (0.3, 1.7)
Asymptomatic person with COVID-19 can transmit the virus			
No	1	1	1
Yes	2.0 (1.2, 3.6)*	1.5 (1.0, 2.5)	1.5 (0.9, 2.5)
COVID-19 can be transmitted among adolescents and young adults			
No	1	1	1
Yes	1.0 (0.7, 1.6)	1.1 (0.7, 1.6)	0.8 (0.6, 1.3)
Avoiding crowdedness in public places can prevent COVID-19			
No	1	1	1
Yes	0.5 (0.1, 2.7)	2.6 (0.4, 17.4)	1.8 (0.3, 10.5)
Covering the nose and mouth while coughing can prevent COVID-19			
No	1	1	1
Yes	0.6 (0.2, 1.8)	1.1 (0.4, 3.4)	1.3 (0.5, 3.6)
Avoiding touching of the eyes, nose, and mouth can prevent COVID-19			
No	1	1	1
Yes	1.6 (0.7, 3.7)	0.7 (0.4, 1.4)	1.1 (0.6, 2.3)
No need for preventive measures; COVID-19 is not deadly			
No	1	1	1
Yes	0.6 (0.4, 1.0) **	0.6 (0.4, 1.0) **	1.1 (0.7, 1.6)
Heard of COVID-19 via an educational/training program			
No	1	1	1
Yes	1.0 (0.6, 1.6)	1.2 (0.8, 1.8)	1.0 (0.6, 1.4)

aOR = adjusted odds ratio. * = significant at *P*-value < 0.05; ** = significant at *P*-value < 0.05, but CI includes the null value which is because of the rounding effect; 1 = reference category.

The odds of handwashing or hand sanitizing among students in the technical program had a 60% lower odds of practicing handwashing or hand sanitizing than students in the science program (aOR: 0.4; 95% CI: 0.2, 0.8). Students who reported that COVID-19 was not deadly had a 40% lower odds of practicing handwashing or hand sanitizing than those who reported that the virus was deadly (aOR: 0.6; 95% CI: 0.4, 1.0; Table 2).

Students who reported that COVID-19 can be transmitted via droplets from the nose or mouth of an infected person had 2.1 times the odds of practicing social distancing, compared with students who reported that the virus was not transmitted via droplets from the nose or mouth of an infected person (aOR: 2.1; 95% CI: 1.0, 4.5) (Table 2).

## DISCUSSION

Our analysis found that at least 50% of the students either did not wear a face mask, practice handwashing/hand sanitizing, or practice social distancing often/always. Students who reported that COVID-19 can be transmitted via droplets from the nose or mouth of an infected person and students who reported that asymptomatic persons can transmit the virus were positively associated with wearing a face mask. However, students who reported that COVID-19 was not deadly were less likely to wear a face mask than those who reported that the virus was deadly. Similarly, students who reported that COVID-19 was not deadly had a lower odds of practicing handwashing or hand sanitizing. It was also revealed that students in the technical program were also less likely to practice handwashing or hand sanitizing than those in the science program. The study also established that students who reported that COVID-19 can be transmitted via droplets from the nose or mouth were positively associated with the practice of social distancing.

The low prevalence of practice of COVID-19 preventive measures in our study might be due to several possible reasons. Many students might not be well informed about the morbidity and mortality associated with COVID-19. Some students might also not be aware of the mode of transmission and the importance of practicing COVID-19 preventive measures. School authorities might also not be enforcing the practice of COVID-19 safety protocols. Schools might also not have adequate hand sanitizers for students or handwashing stations at vantage points on campus. The low prevalence of practice of COVID-19 preventive measures in our study is worrying as this can lead to local outbreaks in schools if a student gets infected.

We found that students who reported that COVID-19 can be transmitted via droplets from the nose or mouth of an infected person were more likely to wear a face mask than those who reported otherwise. This finding is not surprising as students with knowledge that COVID-19 can be transmitted via droplets from the nose or mouth of an infected person might also be aware of the protective effects offered by face mask against droplets,^21^ compared with students who did not know that transmission via droplets was possible. Our findings on the positive association between students who reported that asymptomatic persons can transmit the virus and wearing a face mask might be because students are aware of the recent evidence suggesting that COVID-19 can be transmitted from an asymptomatic person.^22^ We did also observe that students who reported that COVID-19 was not deadly were less likely to wear a face mask than those who reported that the virus was deadly. This finding reflects the lack of knowledge among some students about mortality associated with COVID-19. Although there is the need to educate students on the importance of wearing a face mask, students need to know that COVID-19 can be fatal.

Our study found that students in the technical program were less likely to practice handwashing or hand sanitizing than those in the science program. This finding in our study might be because students in the science program have a better understanding on the importance of handwashing/hand sanitizing in disease prevention than their peers in the technical program. We did also find that students who reported that COVID-19 was not deadly had a lower odds of practicing handwashing or hand sanitizing than those who reported that the virus was deadly. This emphasizes the need for students to understand that COVID-19 can be fatal and the important role handwashing/hand sanitizing plays in COVID-19 prevention. We also found that students who reported that COVID-19 can be transmitted via droplets from the nose or mouth of an infected person were more likely to practice social distancing than those who reported that the virus cannot be transmitted via droplets from the nose or mouth of an infected person. Students who reported that COVID-19 transmission via droplets was possible practiced social distancing as they might be aware that social distancing reduces the virus transmission by droplets.^23,24^

Our study had some limitations. The cross-sectional design of our study does not allow for causal interpretation of our findings. Another limitation is that our primary outcomes were self-reported and were subject to recall bias. However, we expect recall bias to be similar between exposed and unexposed predictor variables. Our primary outcome, social distancing, was also not objectively measured, and was therefore subject to measurement error. Another limitation of our study was the inability to account for school differences. One other limitation is that the true population of students who practiced any of the COVID-19 preventive measures is likely overestimated in our study as students are obliged to observe COVID-19 safety protocols according to the directive by the government of Ghana. Finally, our study took place in selected schools in one of the 16 regions in Ghana; therefore, our findings might not be generalizable to the entire country. Nonetheless, our findings fill an important gap in the literature and provide relevant information on factors associated with the practice of COVID-19 preventive measures among students in Ghana.

## CONCLUSION

This is the first study in Ghana to explore factors associated with practice of COVID-19 preventive measures among senior high schools. Our findings provide guidance to schools on factors that might contribute to ensuring that students of senior high schools in Ghana practice COVID-19 preventive measures. It may also be relevant for COVID-19 health education to be introduced in senior high schools. Our findings may also be relevant to those of senior high schools in low- and middle-income countries contemplating on reopening schools within this pandemic.
